# Impact of Health Policy Changes on Emergency Medicine in Maryland Stratified by Socioeconomic Status

**DOI:** 10.5811/westjem.2017.1.31778

**Published:** 2017-03-13

**Authors:** Laura Pimentel, David Anderson, Bruce Golden, Edward Wasil, Fermin Barrueto, Jon M. Hirshon

**Affiliations:** * University of Maryland School of Medicine, Department of Emergency Medicine, Baltimore, Maryland; † Baruch College, Zicklin School of Business, Operations Management, City University of New York, New York, New York; ‡ University of Maryland, Robert H. Smith School of Business, Management Science, College Park, Maryland; § American University, Kogod School of Business, Department of Information Technology, Washington, DC

## Abstract

**Introduction:**

On January 1, 2014, the financing and delivery of healthcare in the state of Maryland (MD) profoundly changed. The insurance provisions of the Patient Protection and Affordable Care Act (ACA) began implementation and a major revision of MD’s Medicare waiver ushered in a Global Budget Revenue (GBR) structure for hospital reimbursement. Our objective was to analyze the impact of these policy changes on emergency department (ED) utilization, hospitalization practices, insurance profiles, and professional revenue. We stratified our analysis by the socioeconomic status (SES) of the ED patient population.

**Methods:**

We collected monthly mean data including patient volume, hospitalization percentages, payer mix, and professional revenue from January 2013 through December 2015 from a convenience sample of 11 EDs in Maryland. Using regression models, we compared each of the variables 18 months after the policy changes and a six-month washout period to the year prior to ACA/GBR implementation. We included the median income of each ED’s patient population as an explanatory variable and stratified our results by SES.

**Results:**

Our 11 EDs saw an annualized volume of 399,310 patient visits during the study period. This ranged from a mean of 41 daily visits in the lowest volume rural ED to 171 in the highest volume suburban ED. After ACA/GBR, ED volumes were unchanged (95% confidence interval [CI] [−1.58–1.24], p=.817). Hospitalization percentages decreased significantly by 1.9% from 17.2% to 15.3% (95% CI [−2.47%–1.38%], p<.001). The percentage of uninsured patients decreased from 20.4% to 11.9%. This 8.5% change was significant (95% CI [−9.20%–7.80%], p<.001). The professional revenue per relative value unit increased significantly by $3.97 (95% CI [3.20–4.74], p<.001). When stratified by the median patient income of each ED, changes in each outcome were significantly more pronounced in EDs of lower SES.

**Conclusion:**

Health policy changes at the federal and state levels have resulted in significant changes to emergency medicine practice and finances in MD. Admission and observation percentages have been reduced, fewer patients are uninsured, and professional revenue has increased. All changes are significantly more pronounced in EDs with patients of lower SES.

## INTRODUCTION

### Background

On January 1, 2014, the financing and delivery of healthcare in the state of Maryland changed profoundly. Four important provisions of the Affordable Care Act (ACA) were implemented on that day: guaranteed issue of health insurance to all citizens regardless of pre-existing medical conditions; the expansion of access to Medicaid coverage to individuals earning up to 138% of the federal poverty level; the provision of income-based tax credits and subsidies for the purchase of health insurance; and the requirement for all U.S. citizens to obtain qualified health insurance coverage. 1 Ten days later, a major revision to the Maryland Medicare waiver was announced, with the explicit goal of transforming the state’s healthcare delivery system from a volume-based fee-for-service model to a value-based population health model. The new waiver ushered in a global budget revenue (GBR) structure for hospital reimbursement. 2 These two major policy changes substantially and uniquely affected emergency department (ED) finances and clinical operations in Maryland.

The ACA has two overarching objectives. The first is to increase access to healthcare through the establishment of health insurance exchanges and Medicaid programs. The second is to reform the healthcare delivery system so as to decrease the growth rate in spending and improve the quality of care. The first objective has an immediate effect as people matriculate into health insurance exchanges. The second goal is complex and involves mechanisms such as incentivizing reduction in Medicare readmissions, hospital-acquired conditions, and payment structures emphasizing value over volume. 3

The state of Maryland is geographically diverse, with urban, suburban, and rural populations. Between 2011 and 2013, median household incomes ranged from $32,997 in rural Somerset County to $107,452 in suburban Howard County. 4

The Maryland Medicare waiver is the result of legislation passed in 1977, which exempts the state from the Inpatient and Outpatient Prospective Payment Systems. It also allows the state’s Health Services Cost Review Commission (HSCRC) to set hospital rates that Medicare and all other insurance companies must pay. 5 Important goals of the all-payer concept are to distribute the burden of uncompensated care throughout the state, provide robust support for graduate medical education, and control costs. The waiver was contingent upon keeping the cost per Medicare admission below the national average. The waiver revision was necessary because at that time the total hospital costs per Medicare beneficiary had grown significantly in Maryland. In 2014, the revised waiver created an all-payer global budget model that caps total hospital revenue growth at rates related to the gross state product and converted hospital reimbursement from a volume-based model to a value-based model. Under GBR the hospital’s margin is the difference between the global budget cap and actual expenses. Each admission no longer improves the hospital’s bottom line. To increase margins, hospitals have to manage the health of the populations they serve in the lowest cost settings and minimize expenditures associated with hospital stays. To maintain the waiver, Maryland must reduce the rate of growth of hospital costs per Medicare beneficiary below the national average. Consistent with the ACA, other metrics of success include reductions in the incidence of hospital-acquired conditions and the number of Medicare readmissions. 5 Health policy experts at the Centers for Medicare and Medicaid Services (CMS) and in Maryland anticipate that the success of the new Maryland waiver will serve as a national model for other states interested in an all-payer system.^2^,^6^,^7^

**Population Health Research Capsule**

What do we already know about this issue?

The insurance provisions of the Affordable Care Act were implemented in 2014. Maryland revised its Medicare waiver in 2014 creating a Global Budget Revenue model for acute care hospitals.

What was the research question?

How did these federal and state policy changes affect emergency department volumes, payer mix, hospitalizations, and finances?

What was the major finding of the study?

Volumes were unchanged; rates of uninsured patients decreased; hospitalization percentages decreased; revenue increased.

How does this improve population health?

Increased percentages of emergency patients have insurance and receive care in outpatient settings. These findings were greater in practices serving patients of lower socioeconomic status.

### Importance

Emergency physicians (EP) have a critical role in healthcare utilization, as they make or participate in decisions regarding the disposition of more than half of all patients admitted to acute care hospitals. 8 Because of this integral role in hospital patient care and resource utilization, it is clear that major policy changes affecting hospitals have substantial impact on ED practice.

### Goals of This Investigation

Our primary objective was to study the impact of the ACA and GBR on ED utilization, insurance profiles, professional reimbursement, and hospitalization practices in Maryland. We stratified our analysis by the socioeconomic status (SES) of each ED population involved in this analysis to better understand the differential impact of these changes. We hypothesized that the impact of policy changes would be more pronounced in EDs located in lower SES communities.

## METHODS

### Study Design

We performed a retrospective pre/post-intervention study with a washout period.

### Study Population

We examined a convenience sample of 11 EDs in Maryland, representing a cross-section of locations, sizes, and median incomes. Our study sites ranged from low-volume rural EDs to urban academic EDs. The rural sites are three EDs located on the Eastern Shore of MD. One of the three is a freestanding facility. The urban EDs are located in Baltimore City. One is a large academic institution. Two are lower volume inner-city EDs. One of the study sites is a large county ED located in a Washington, DC, suburb. Our suburban study sites are located in northern and central MD. One is a freestanding facility. Using regression models and before-and-after comparisons, we analyzed the impact of new health financing policies on Maryland’s EDs.

### Data Source and Management

We collected monthly volume and admissions data from the health information systems of the 11 EDs. Revenue and payer-mix data were obtained from monthly billing company reports. We analyzed data from January 2013 to December 2015 (encompassing the 12 months preceding the January 1, 2014 ACA/GBR implementation and the subsequent 24 months). For our analysis, we considered the six-month period from January 1, 2014 through June 30, 2014 a washout period. Our study compared the 18 months from July 1, 2014, through December 31, 2015, to calendar year 2013. Collected information included visit volume, hospitalization defined as the combined admission/observation rate, revenue per relative value unit (RPRVU), and payer mix (percent uninsured, percent Medicaid, percent private insurance, percent Medicare).

We defined visit volume as the total number of registered ED visits in each study site. This number was collected monthly from each ED’s information system and divided by the number of days in the month and reported as mean visits per day. We calculated the hospitalization rate by taking the sum of the number of ED patients admitted to the hospital or placed in an observation status and dividing that total by the number of ED visits for the month.

The RPRVU reflects professional revenue. In the study practices, the professional coding is done by trained coders who assign evaluation and management levels and procedure codes based on provider documentation. The RVUs are calculated from the codes based on the Center for Medicare and Medicaid’s RVU weighting for each code. The RPRVU is a calculation based on total charges for a given month multiplied by the estimated collection percentage for each practice and divided by the total number of RVUs. The estimated collection percentage reflects historical experience with that practice.

We performed the payer-mix calculations by taking the total number of visits associated with each insurance category per month and dividing that number by the total number of visits for the month.

In our freestanding EDs, the hospitalization volume was calculated from the number of patients transferred to area hospitals for inpatient care. We calculated the median income of each ED’s catchment area, using 2010 census data for ZIP code income. The study was considered non-human subjects research, which does not require institutional review board approval at our institution.

### Data Analysis

We used multiple regression models to determine the effect of ACA/GBR implementation on hospital financial and operational performance. Outcome measures were regressed on a binary indicator variable that indicated whether or not ACA/GBR had been implemented. We controlled for differences between hospitals by including a set of dummy variables for each of them. The regression equation used for each outcome has the form –

$$\text{Outcome}=\beta_{0}+\beta_{1}\;\text{ACA}+\beta \;\text{Facility}$$

-- where Outcome is the outcome of interest (e.g., RPRVU, admission rate, un-insurance rate, etc.), β_0_ is the intercept, β_1_ is the estimated effect for the ACA implementation, ACA is an indicator variable that is 1 in months January 2014 and after, and 0 before, β is a vector of coefficients for each ED, and Facility is a vector of facility indicator variables.

To ensure that the results we obtained were not simply the continuation of pre-existing trends, we regressed the outcome variables on the baseline year of data, calendar year 2013, for each of the outcomes of interest. We then compared the outcomes in 2014 to what the value would have been had the 2013 trends continued. In most cases the 2013 trends were small, so differences were not significant.

To examine the potential differential impact of ACA/GBR implementation, we explored whether the SES of the patient population was an effect modifier. For this analysis, we used the estimated median income of each ED’s catchment area. For each site, we recorded the 10 ZIP codes with the highest percentages of patients and the percent of patients from each of those ZIP codes. We computed a weighted average of the median income from the 2010 U.S. census of each of those ZIP codes to produce a measure of the median income for the patient population for each ED. We included the median income of the ED population as an explanatory variable and interacted it with ACA/GBR implementation to seek differences in ED outcomes based on the income of the catchment area. When SES is included, the regression equation becomes –

$$\text{Outcome}=\beta_{0}+\beta_{1}\;\text{ACA}+\beta_{2}\;\text{ACA Median Income}+\beta \;\text{Facility}$$

-- where Median Income is the weighted average of the median income of the catchment area and β_2_ is the interaction effect.

## RESULTS

The 11 EDs saw an annualized volume of 399,310 visits during 2013 through 2015, ranging from a mean of 41 daily visits in the lowest-volume rural ED to 171 in the highest-volume suburban ED.

**Figure 1:**
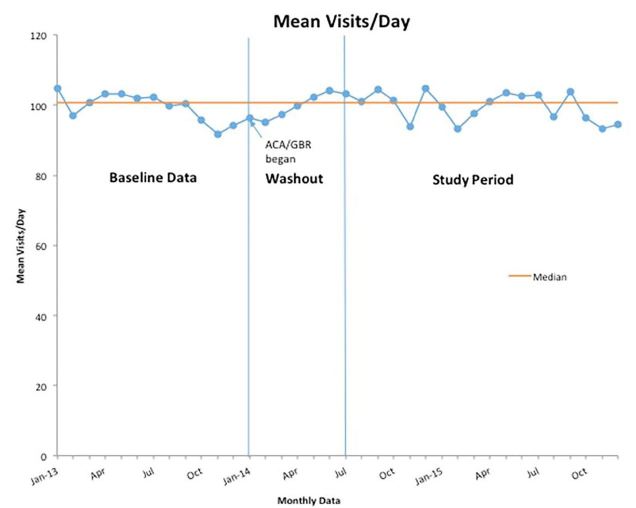
Run chart of monthly mean emergency department visits per day from baseline year through study period. *ACA,* Affordable Care Act; *GBR,* global budget revenue.

With regard to number of ED visits over the study period before and after the policy changes, our regression analysis found no significant relationship between ACA/GBR implementation and ED volume (Figure 1, Table 1s). The average volume per hospital went down by .17 patients per day per site (95% confidence interval [CI] [−1.58, 1.24], p=.817). However, before the policy change there had been a small volume decrease that flattened out after. As a result, the relative increase in ED volume of 16.6 (15.184, 17.954) patients reached statistical significance on a trend-adjusted basis. (Table 2s).

**Figure 2:**
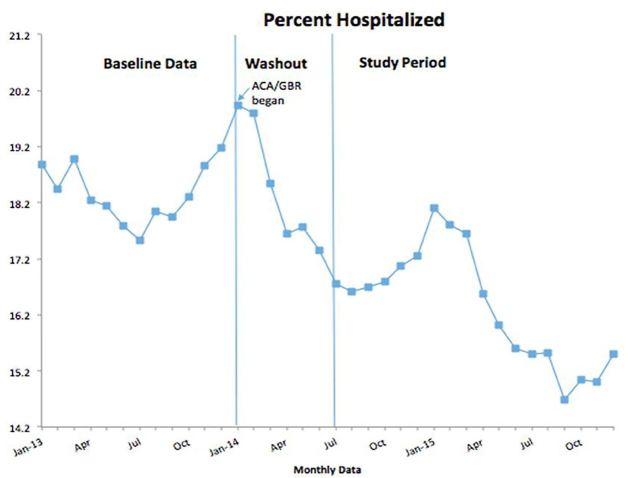
Run chart of the percentage of patients hospitalized from baseline year through study period. *ACA,* Affordable Care Act; *GBR,* global budget revenue.

In an analysis of the impact of ACA/GBR implementation on the percentage of patients hospitalized, we found that rates decreased significantly after July 1, 2014 (95% CI: (−1.80%, −0.80%), p<.001) (Figure 2, Table 3s). When controlling for the pre-implementation trend, the decrease is still statistically significant (95% CI [−2.47%, −1.38%], p<.001). The admission rate was 1.9 percentage points lower than in the previous year. The mean hospitalization rate dropped from 17.2% to 15.3%, an 11% relative reduction.

**Figure 3:**
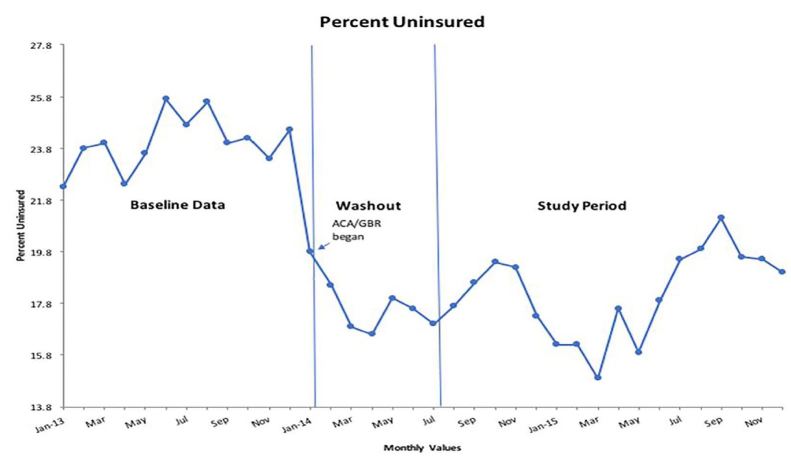
Run chart of the percentage of uninsured patients from baseline year through study period. *ACA,* Affordable Care Act; *GBR,* global budget revenue.

Our analysis of the percentage of uninsured ED patients before and after the implementation of the ACA/GBR is given in Figure 3 and Table 4s. The rate of uninsured patients decreased by a statistically significant 8.5 percentage points (95% CI [−9.20%, −7.80%], p<.001). Before implementation of the ACA, the average ED month had 20.4% uninsured patients. After implementation, the rate was 11.9%, a relative reduction of 42%. The percentage of patients covered by Medicaid increased by 8.5% (95% CI [7.7%, 9.2%], p<.001), the percentage covered by Medicare increased by 0.9% (95% CI [0.6%, 1.2%], p<.001), and the percentage with private insurance decreased by 1.9% (95% CI [−2.5%, −1.2%], p<.001).

**Figure 4:**
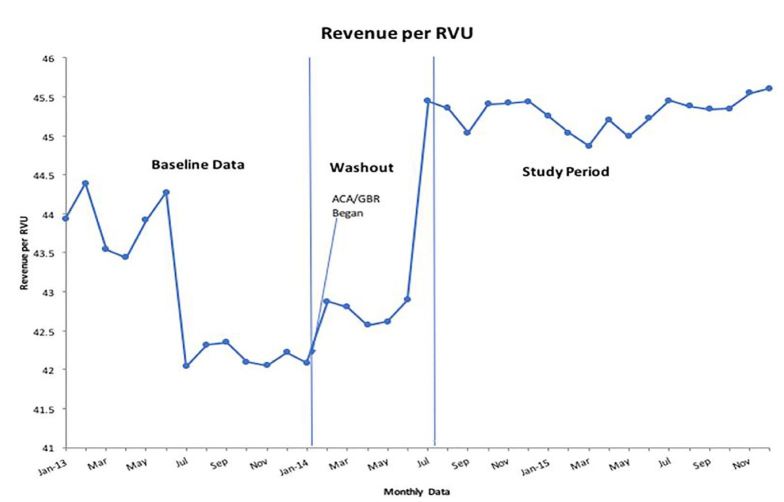
Run chart of the revenue per relative value unit from baseline year through study period. *RVU*, Relative Value Unit

Regression analysis of the professional RPRVU over the study period shows a mean increase of $3.97 (95% CI [3.20, 4.74], p<.001) after implementation of the ACA/GBR as seen in Figure 4 and Table 5s. This increase represents a statistically significant 10.7% change.

An alternative explanation for the fact that we see changes in outcomes after January 1, 2014, is that there is a preexisting trend that simply continues throughout the entire observation period. Looking specifically at the baseline period, the 12 months prior to implementation of the policy changes, we found no statistically significant trends in either revenue per RVU (95% CI [−0.14, 0.22], p=0.65), the percent uninsured (95% CI [−0.01, 0.20], p=0.07), or percent admitted (95% CI [−.002, 0.12], p=.06). Regardless, we ran the regressions again, correcting for these possible underlying trends, shown in Table 2s. Although not statistically significant, in the case of uninsured rate and admission rates, the trend that we see is in the opposite direction of the effect observed after January 1, 2014. If anything, our estimates of the effects are underestimating the true underlying effect. We did see one significant trend in 2013: ED volume was decreasing. This trend flattened during the study period.

**Figure 5:**
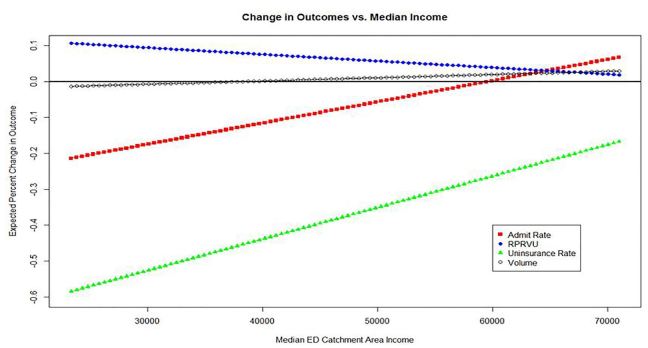
Expected percent changes in outcome vs. median ED income. *ED*, Emergency department; *RPVU,* revenue per relative value unit.

Turning to the moderating effect of SES on our results, we found that the interaction of median catchment area income and ACA/GBR implementation was statistically significant in each model. The median annual incomes of the catchment areas of the 11 EDs ranged from a low of $22,900 to a high of $70,000 (Table). The changes in each outcome are more pronounced for ED populations with lower median incomes. Figure 5 shows the expected change in outcome for an ED of a given income level. A 57% decrease in the uninsured rate is expected at an ED with a catchment area median income of $25,000, but only a 22% decrease at one with a median income of $70,000. The lower the income of the catchment area, the greater the expected increase in RPRVU. We estimated a 10% increase in RPRVU for a hospital with a catchment area median income of $25,000 but predicted no change at an ED with a median income of $70,000. Admission rates decreased the most at poorer hospitals as well, ranging from a decrease of 22% to no significant change.

## DISCUSSION

Our study reports on the impact of the ACA and GBR policy changes implemented simultaneously at the state and federal levels, on EDs in Maryland. We found that ED volumes experienced a small, significant increase only on a trend-adjusted basis. Hospitalizations significantly decreased and the percentage of patients with insurance significantly increased, as did professional revenue.

A stated goal of both the ACA and GBR was to reduce the number of ED visits. 9–11 During the first 18 months of the new policies, we found minimal change in the volumes of patients using emergency services in Maryland. In contrast, after insurance coverage was expanded in Oregon and Massachusetts, ED use increased, particularly during the first-year transition from no insurance to Medicaid coverage.^12^,^13^ Because of GBR, unique to Maryland, it is possible that newly insured patients are receiving more care in settings such as urgent care centers, outpatient clinics, physicians’ offices, and patient-centered medical homes.^14^,^15^

The structure of the new policies in the federal healthcare exchanges is another reason that the ACA may result in lower utilization of healthcare services, including the ED. High deductibles and co-payments are features of the plans with the lowest premiums. The lowest-cost bronze plans have annual deductibles that exceed $5,000 for individuals and $10,000 for families. In contrast, deductibles in employer-provided insurance plans average $1,135. These high out-of-pocket costs might have had a suppressive effect on ED utilization particularly among patients transitioning from plans with lower first-dollar costs. 16

Examining the impact of policy changes on hospitalization practices, we found that EPs in Maryland decreased their use of inpatient resources by an absolute 1.3% and a relative decrease of 8.2%. An analysis of one large multi-state nonprofit hospital system, which compared hospital admissions before and after implementation of the ACA coverage expansions in 2014, showed a relative decrease of 2.4% in hospital admissions across the system. 17 However, striking differences by payer were evident. Medicaid admissions increased by 7.4% in Medicaid expansion states and by 1.4% in non-expansion states. This suggests that the significant decline in inpatient utilization by ED patients in Maryland, a Medicaid expansion state, is more heavily influenced by GBR than ACA. 18 When examining the data in relation to SES, we noted a significantly greater impact on less-affluent patient populations (Figure 5).

Maryland EP groups have been important partners with hospitals in striving for success under GBR. This partnership is critically important, because EPs have a direct impact on half of all hospital admissions. 8 The design and implementation of care plans for high utilizers of ED services are showing promising results with respect to decreasing hospital admissions, observations, and resource utilization.^19^,^20^

Another important approach is the application of evidence-based risk-stratification tools designed to decrease variations in EP practices, a source of potentially avoidable utilization (PAU).^21^,^22^ These tools include the Pneumonia Severity Index and its associated Pneumonia Outcomes Research Trial (PORT) score. 23 The work of Peterson and colleagues on the identification of high-risk characteristics of patients with soft-tissue infections anticipates the development of a risk stratification tool. 24 An EP group in Maryland has taken the lead in implementing the HEART score, a protocol that uses the validated prediction rule for low-risk chest pain patients. This score has proven to be a powerful tool for decreasing variation in physician practice and minimizing PAU. 25–27 Similarly, Maryland EPs have incorporated the Choosing Wisely guidelines compiled by the American College of Emergency Physicians into their practices. 28 Emphasis has also been placed on adherence to guidelines for the workup of patients in whom pulmonary emboli are suspected, using a framework that incorporates pulmonary embolism rule-out criteria (PERC) and the stratification of patients into low-, medium-, and high-risk categories. 29–31 We surmise that the increased use of observation status for short-stay patients is the result of EPs’ attempts to decrease admission/readmission rates, in accordance with CMS payment policies; this trend has been observed elsewhere in the country.^32^

With respect to the greater impact of policy changes on less affluent communities, hospitals and health systems have been incentivized by the GBR structure to meaningfully improve access to outpatient resources and follow-up care. Examples include the establishment of a wound and soft-tissue clinic that can be used for follow-up appointments by all ED patients with skin pathology, regardless of their insurance status. Enhanced mechanisms that expedite patient follow-up with primary care, cardiology, orthopedics, and mental health practices or clinics have been developed. These include the ability of ED personnel to schedule specific expedited appointments around the clock without having to page or call the referral office or provider. The increase in the number of patients with insurance coverage improves the financial viability of these new endeavors. Newly insured patients now have access to resources once available only to more affluent populations.

We found a statistically significant improvement in the insurance profile of ED patients in Maryland. Most of the change can be attributed to the transition of previously uninsured patients to Medicaid coverage. There was a spectrum of outcomes, with the greatest changes in EDs with the lowest SES and the least significant changes in the most affluent communities. Similarly, the RPRVU increased significantly more in the low SES practices. These financial improvements are directly attributable to the ACA. It is important to note that GBR is strictly a hospital initiative at this time and does not include physician revenue. Revenue improvements have been particularly important in Maryland, where physician reimbursement from insurance companies has been notably below national averages. 33 These increases will lead to better physician coverage in these EDs and lower reliance on hospital subsidies. This directly decreases disparities in coverage and care between EDs of higher and lower SES.

## LIMITATIONS

Our study is based on a convenience sample of Maryland EDs. According to the 2014 Health Services Cost Review Commission report on ED visits in Maryland, the patient volume of the 11 departments in this study constitutes 16% of the total ED visits in the state. The EDs in this study constitute a cross-section of urban, suburban, and rural locations practicing academic and community medicine. The median income of the communities ranged from just under $23,000 to just over $70,000. Nevertheless, this sample might not be completely representative of the experience of all EDs in the entire state. Similarly, the median income of a community may not represent the SES of those using emergency services.

Maryland is geographically one of the smallest states in the country. Located in the mid-Atlantic, the state has a population of nearly six million residents. The largest city, Baltimore, has 620,000 residents. It is not clear that the impact of health policy in Maryland is generalizable to other states, particularly those with substantially larger cities and different demographics.

Because the ACA and GBR were implemented simultaneously, it is difficult to separate the impact of the federal program from the state program. Our study was not designed to specifically attribute the changes in ED practice to one policy or the other. We did not study clinical outcomes in this analysis and cannot relate increased insurance coverage or decreased hospitalization to the quality of care provided.

Our analysis is an early look at the ramifications of significant policy changes. Initiatives of this magnitude might require longer time frames to achieve policy goals. It is certainly possible that ED volumes and hospitalization percentages will change as hospitals and health systems continue to transition to population health. It will be important to continue to analyze the system as patients’ use patterns change based on their access to insurance and resources.

We looked at a select number of outcome measures in our analysis. Other important effects of ACA/GBR are also worthy of analysis to attain a more complete understanding of the impact on Maryland ED patients. We strongly believe that continued research is indicated.

## CONCLUSION

The simultaneous implementation of health policy changes at the federal and state levels in Maryland is changing the practice of emergency medicine. Fewer patients are now admitted to hospitals or observation units. The percentage of uninsured patients has decreased associated with increased professional revenue. All changes are significantly greater in practices serving populations of lower SES. Further research on the impact of these changes on clinical operations and patient outcomes is warranted.

## Supplementary Information











## Figures and Tables

**Table:** Emergency department (ED) and median income weighted by ED catchment Zip codes.

Hospital	Income
Hospital A	$23,616
Hospital B	$70,041
Hospital C	$56,337
Hospital D	$45,808
Hospital E	$31,192
Hospital F	$40,242
Hospital G	$56,716
Hospital H	$22,909
Hospital I	$58,028
Hospital J	$45,556
Hospital K	$26,307
