# Supplementary material for: Impact of Health Policy Changes on Emergency Medicine in Maryland Stratified by Socioeconomic Status
Source: West J Emerg Med. 2017 Mar 13;18(3):356–65. doi: 10.5811/westjem.2017.1.31778 (PMC5391884; doi:10.5811/westjem.2017.1.31778)
Supplement: Supplementary file 1 [file wjem-18-356-s001.pdf]

Table 1s. ED volume (visits per day) regressed on ACA/GBR implementation and hospital

Variable	Estimate	Std. Error	t-value	95% CI	p-value
ED A	74.011	1.243	59.5	(71.575 , 76.446)	<.0001
ED B	100.444	1.243	80.8	(98.008 , 102.879)	<.0001
ED C	90.503	1.243	72.8	(88.068 , 92.939)	<.0001
ED D	127.833	1.243	102.8	(125.398 , 130.269)	<.0001
ED E	58.476	1.243	47.1	(56.041 , 60.912)	<.0001
ED F	95.862	1.243	77.1	(93.426 , 98.297)	<.0001
ED G	41.286	1.243	33.2	(38.850 , 43.721)	<.0001
ED H	87.276	1.243	70.2	(84.841 , 89.712)	<.0001
ED I	172.241	1.243	138.6	(169.806 , 174.676)	<.0001
ED J	80.879	1.243	65.1	(78.444 , 83.315)	<.0001
ED K	165.785	1.257	131.9	(163.321 , 168.249)	<.0001
Summary	-0.166	0.720	-0.2	(-1.577 , 1.244)	0.817

ED, Emergency department; *ACA,* Affordable Care Act; *GBR,* Global Budget Revenue; *Summary*, Summary of ACA/GBR Impact on ED volume (visits per day)
