# Supplementary material for: Impact of Health Policy Changes on Emergency Medicine in Maryland Stratified by Socioeconomic Status
Source: West J Emerg Med. 2017 Mar 13;18(3):356–65. doi: 10.5811/westjem.2017.1.31778 (PMC5391884; doi:10.5811/westjem.2017.1.31778)
Supplement: Supplementary file 2 [file wjem-18-356-s002.pdf]

Table 2s. ED volume (visits per day) regressed on ACA/GBR implementation and hospital with trend-adjusted comparisons

Variable	Estimate	Std. Error	t-value	95% CI	p-value
ED A	78.322	1.220	64.2	(75.931 , 80.713)	<.0001
ED B	104.755	1.220	85.9	(102.364 , 107.146)	<.0001
ED C	94.815	1.220	77.7	(92.424 , 97.206)	<.0001
ED D	132.145	1.220	108.3	(129.753 , 134.536)	<.0001
ED E	62.787	1.220	51.4	(60.396 , 65.178)	<.0001
ED F	100.173	1.220	82.1	(97.782 , 102.564)	<.0001
ED G	45.597	1.220	37.4	(43.206 , 47.988)	<.0001
ED H	91.587	1.220	75.1	(89.196 , 93.978)	<.0001
ED I	176.552	1.220	144.7	(174.161 , 178.943)	<.0001
ED J	85.191	1.220	69.8	(82.800, 87.582)	<.0001
ED K	169.862	1.234	137.6	(167.442 , 172.281)	<.0001
Summary	16.569	0.707	23.5	(15.184 , 17.954)	<.0001

ED, Emergency department; *ACA,* Affordable Care Act; *GBR,* Global Budget Revenue; *Summary,* Summary of ACA/GBR Impact on ED volume with trend-adjusted comparison
