# Supplementary material for: Impact of Health Policy Changes on Emergency Medicine in Maryland Stratified by Socioeconomic Status
Source: West J Emerg Med. 2017 Mar 13;18(3):356–65. doi: 10.5811/westjem.2017.1.31778 (PMC5391884; doi:10.5811/westjem.2017.1.31778)
Supplement: Supplementary file 3 [file wjem-18-356-s003.pdf]

Table 3s. Percentage hospitalized from the emergency department regressed on ACA/GBR implementation and hos pital.

Variable	Estimate	Std. Error	t-value	95% CI	p-value
ED A	22.309	0.476	46.8	(21.376 , 23.242)	<.0001
ED B	7.330	0.485	15.1	(6.379 , 8.280)	<.0001
ED C	15.559	0.476	32.7	(14.625 , 16.492)	<.0001
ED D	18.852	0.485	38.9	(17.902 , 19.803)	<.0001
ED E	12.631	0.476	26.5	(11.698 , 13.564)	<.0001
ED F	17.858	0.476	37.5	(16.925 , 18.792)	<.0001
ED G	6.205	0.476	13.0	(5.272 , 7.139)	<.0001
ED H	20.715	0.476	43.5	(19.781 , 21.648)	<.0001
ED I	26.709	0.476	56.1	(25.776 , 27.642)	<.0001
ED J	22.976	0.482	47.7	(22.031 , 23.920)	<.0001
ED K	29.870	0.482	62.0	(28.925 , 30.814)	<.0001
Summary	-1.924	0.277	-6.9	(-2.467 , -1.380)	<.0001

ACA, Affordable Care Act; *CI,* confidence interval; *ED*, emergency department; *GBR*, Global Budget Revenue; *Summary,* Summary of ACA/GBR Impact on Percentage hospitalized from ED
