# Supplementary material for: Impact of Health Policy Changes on Emergency Medicine in Maryland Stratified by Socioeconomic Status
Source: West J Emerg Med. 2017 Mar 13;18(3):356–65. doi: 10.5811/westjem.2017.1.31778 (PMC5391884; doi:10.5811/westjem.2017.1.31778)
Supplement: Supplementary file 4 [file wjem-18-356-s004.pdf]

Table 4s. Fraction of uninsured patients regressed on ACA/GBR implementation and hospital

Variable	Estimate	Std. Error	t-value	95% CI	p-value
ED A	0.259	0.006	40.9	(0.247 , 0.272)	<.0001
ED B	0.175	0.006	27.6	(0.163 , 0.187)	<.0001
ED C	0.256	0.006	40.4	(0.244 , 0.269)	<.0001
ED D	0.302	0.006	47.6	(0.289 , 0.314)	<.0001
ED E	0.176	0.006	27.7	(0.163 , 0.188)	<.0001
ED F	0.159	0.006	25.0	(0.146 , 0.171)	<.0001
ED G	0.151	0.006	23.8	(0.139 , 0.163)	<.0001
ED H	0.210	0.006	33.1	(0.197 , 0.222)	<.0001
ED I	0.143	0.006	22.6	(0.131 , 0.156)	<.0001
ED J	0.177	0.006	27.9	(0.164 , 0.189)	<.0001
ED K	0.239	0.006	37.6	(0.226 , 0.251)	<.0001
Summary	-0.085	0.004	-23.2	(-0.092 , -0.078)	<.0001

ACA, Affordable Care Act; *GBR*, Global Budget Revenue; *Summary,* Summary of ACA/GBR Impact on Fraction of uninsured patients
