# Supplementary material for: Impact of Health Policy Changes on Emergency Medicine in Maryland Stratified by Socioeconomic Status
Source: West J Emerg Med. 2017 Mar 13;18(3):356–65. doi: 10.5811/westjem.2017.1.31778 (PMC5391884; doi:10.5811/westjem.2017.1.31778)
Supplement: Supplementary file 5 [file wjem-18-356-s005.pdf]

Table 5s. RPRVU regressed on ACA/GBR implementation and hospital

Variable	Estimate	Std. Error	t-value	95% CI	p-value
ED A	29.682	0.689	43.0	(28.331 , 31.033)	<.0001
ED B	50.269	0.677	74.2	(48.943 , 51.596)	<.0001
ED C	42.004	0.677	62.0	(40.677 , 43.330)	<.0001
ED D	34.292	0.677	50.6	(32.966 , 35.618)	<.0001
ED E	33.162	0.677	49.0	(31.835 , 34.488)	<.0001
ED F	35.974	0.677	53.1	(34.648 , 37.301)	<.0001
ED G	42.108	0.677	62.2	(40.782 , 43.434)	<.0001
ED H	29.422	0.677	43.4	(28.096 , 30.748)	<.0001
ED I	47.797	0.677	70.6	(46.470 , 49.123)	<.0001
ED J	35.570	0.677	52.5	(34.243 , 36.896)	<.0001
ED K	28.615	0.685	41.7	(27.273 , 29.957)	<.0001
Summary	3.970	0.393	10.1	(3.200 , 4.740)	<.0001

RPRVU, Revenue per Relative Value Unit; *ACA,* Affordable Care Act; *GBR*, Global Budget Revenue; *Summary,* Summary of ACA/GBR Impact on RPRVU
